# Machine Learning-Based Comparison of Non-Contrast ASL and DSC-MRI Perfusion for Differentiating Recurrent High-Grade Glioma from Treatment Effects

**DOI:** 10.3390/jcm15176505

**Published:** 2026-08-22

**Authors:** Seyit Erol, Halil Özer, Abdussamet Batur, Mehmet Sedat Durmaz, Abidin Kılınçer, Emine Uysal, Hakan Cebeci

**Affiliations:** Department of Radiology, Faculty of Medicine, Selcuk University, Konya 42130, Türkiye

**Keywords:** arterial spin labeling, dynamic susceptibility contrast, high-grade glioma, perfusion MRI, tumor recurrence

## Abstract

**Background/Objectives:** Differentiating high-grade glioma recurrence from treatment-related changes remains challenging on conventional MRI. This study evaluated non-contrast arterial spin labeling (ASL) perfusion MRI for this distinction and compared its performance with dynamic susceptibility contrast (DSC) perfusion MRI. **Methods:** Postoperative follow-up MRI examinations obtained between November 2019 and May 2021 were retrospectively reviewed. The cohort included 63 MRI examinations from 36 adults treated for high-grade glioma. ASL, routine MRI, and DSC images were independently assessed by two neuroradiologists. Final diagnosis was based on histopathology or longitudinal clinical and imaging follow-up. Reader agreement, diagnostic performance, and an exploratory patient-level grouped machine learning analysis using ASL-only, DSC-only, and combined ASL–DSC features were evaluated. **Results:** ASL- and DSC-derived perfusion parameters were significantly higher in tumor recurrence than in treatment-related changes (all *p* < 0.001). Both techniques showed high diagnostic performance; DSC achieved the highest accuracy, whereas ASL provided high specificity across readers. Inter-reader agreement ranged from substantial to almost perfect. In grouped cross-validation, ASL-only, DSC-only, and combined models achieved mean AUCs of 0.946, 0.997, and 0.997, respectively. Permutation testing confirmed that combined-model performance exceeded chance expectations (empirical *p* = 0.002). **Conclusions:** Non-contrast ASL perfusion showed diagnostic performance comparable to DSC for differentiating high-grade glioma recurrence from treatment effects. ASL may provide a reliable non-invasive alternative for longitudinal surveillance, particularly when gadolinium administration is undesirable or contraindicated.

## 1. Introduction

Gliomas are among the most common neoplasms of the central nervous system, accounting for approximately 40–50% of all primary brain tumors and 2–3% of all cancers worldwide. Despite substantial advances in contemporary treatment strategies—including surgery, chemotherapy, and radiotherapy—high-grade gliomas remain a major therapeutic challenge because of their aggressive biological behavior and resistance to conventional treatments [1,2].

Magnetic resonance imaging (MRI) plays a central role in the diagnosis, postoperative assessment, and longitudinal follow-up of patients with gliomas. Current guidelines recommend MRI within the first 48–72 h after surgery and 2–8 weeks following radiotherapy, followed by regular surveillance imaging every 2–4 months during the first three years and every 3–6 months thereafter [3]. However, conventional MRI sequences are often insufficient for reliably differentiating tumor recurrence from treatment-related changes, such as radiation necrosis or pseudoprogression, underscoring the need for advanced imaging techniques.

Arterial spin labeling (ASL) is a noninvasive perfusion imaging technique that does not require intravenous gadolinium-based contrast agents [4]. ASL quantifies cerebral blood flow by magnetically labeling arterial water protons and tracking their inflow into the brain parenchyma [5]. The absence of contrast administration has made ASL an attractive alternative advanced MRI technique, particularly in pediatric patients, individuals with renal insufficiency, and those at risk for nephrogenic systemic fibrosis [6]. Previous studies have demonstrated the utility of ASL in the evaluation of brain tumors, cerebrovascular disorders, and neurodegenerative diseases [7,8,9,10].

Dynamic susceptibility contrast (DSC) perfusion imaging, in contrast, is one of the most widely used advanced MRI techniques in routine clinical practice and enables assessment of the cerebral microvascular environment through intravenous gadolinium administration [1]. Although DSC perfusion provides robust hemodynamic information, repeated contrast-enhanced MRI examinations during postoperative follow-up of patients with high-grade glioma may pose clinical challenges. Consequently, establishing a reliable non-contrast perfusion imaging alternative could substantially improve long-term surveillance strategies in this patient population.

Although ASL has shown promising results in the evaluation of brain tumors, direct comparisons between non-contrast ASL and DSC perfusion MRI in the postoperative follow-up setting of high-grade gliomas remain limited. Moreover, the inter-reader reproducibility and the combined diagnostic value of these perfusion techniques in routine longitudinal surveillance have not been fully clarified. Therefore, the present study aimed to compare the diagnostic performance of non-contrast ASL and DSC perfusion MRI in differentiating tumor recurrence from treatment-related changes in postoperative high-grade glioma follow-up. In addition to conventional qualitative and quantitative analyses, we also performed an exploratory patient-level grouped machine learning analysis to assess the combined diagnostic value of these perfusion parameters. By evaluating both imaging modalities within the same postoperative cohort, this study provides clinically relevant evidence supporting ASL as a reliable non-contrast alternative in longitudinal glioma surveillance.

## 2. Materials and Methods

This retrospective observational study was approved by the Ethics Committee of Selcuk University Faculty of Medicine at its meeting held on 10 March 2021 (approval number: 2021/127) and was conducted in accordance with the Declaration of Helsinki and Good Clinical Practice guidelines. The study period refers to the dates of routine clinical postoperative follow-up MRI examinations performed between November 2019 and May 2021, which were retrospectively identified from the institutional archive and analyzed after ethics approval. No prospective patient recruitment, randomization, treatment allocation, or research-specific imaging intervention was performed. The requirement for informed consent was waived due to the retrospective nature of the study. The requirement for informed consent was waived due to the retrospective nature of the study.

### 2.1. Patient Selection

Between November 2019 and May 2021, adult patients (≥18 years) with a history of surgical resection for high-grade glioma who underwent ASL perfusion imaging in addition to routine postoperative MRI follow-up were retrospectively reviewed. Data on extent of resection were available for all patients and were categorized as gross total resection or subtotal resection; these variables were used for descriptive purposes only. KPS/ECOG scores were not consistently available in this retrospective cohort and were therefore not included. Only patients with tumors classified according to the 2021 WHO Classification of Tumors of the Central Nervous System as glioblastoma, IDH-wildtype, CNS WHO grade 4 or astrocytoma, IDH-mutant, CNS WHO grade 3 were included. Three examinations were excluded due to inadequate image quality. The final study cohort consisted of 63 postoperative MRI examinations from 36 patients, analyzed on an examination-by-examination basis, with each MRI study evaluated independently of the patients’ other MRIs for the presence of tumor recurrence or treatment-related changes.

### 2.2. MRI Acquisition Protocol

All MRI examinations were performed on a 3.0-T MRI system (Siemens Healthineers, Skyra, Erlangen, Germany) using a 32-channel head coil, with patients in the supine position. A gadolinium-based contrast agent (0.1 mmol/kg; Magnevist, Bayer Inc., Leverkusen, Germany) was administered intravenously at a rate of 4 mL/s.

The imaging protocol included axial T1-weighted (TR/TE = 548/8 ms, slice thickness = 4 mm), axial T2-weighted (TR/TE = 5400/80 ms, slice thickness = 4 mm), 3D FLAIR (TR/TE = 4870/394 ms, slice thickness = 0.89 mm), and contrast-enhanced axial and 3D T1-weighted sequences.

ASL perfusion imaging was acquired using a 3D pulsed labeling technique with a combined gradient and spin echo (GRASE) readout (slice thickness = 3 mm, interslice gap = 1.5 mm, FOV = 240 × 220 mm, matrix = 64 × 64, TR/TE = 4600/16 ms, voxel size = 1.5 × 1.5 × 3 mm, bolus duration = 700 ms, inversion time = 1990 ms, total acquisition time = 4 min 59 s).

DSC perfusion imaging was obtained after contrast administration (TR/TE = 1720/30 ms, slice thickness = 5 mm, matrix = 80 × 80, FOV = 220 × 220 mm, acquisition time = 1 min 42 s).

### 2.3. Image Analysis and Qualitative Assessment

All MRI examinations were independently evaluated by two neuroradiologists with 11 and 8 years of experience in neuro-oncologic imaging, respectively. Image analysis was performed using a dedicated workstation (Syngo Via VB30; Siemens Healthineers, Erlangen, Germany).

For ASL imaging, non-contrast conventional MRI sequences and ASL perfusion images were first reviewed. The presence or absence of tumor recurrence was recorded based on areas demonstrating visually increased perfusion. After a washout period of at least two months, DSC perfusion and contrast-enhanced images were evaluated in a separate session without access to the ASL findings.

### 2.4. Quantitative ASL and DSC Analysis

Regions of interest (ROIs) were manually drawn using a freehand technique over hyperperfused areas corresponding to T2-FLAIR hyperintense regions. All ROI measurements were performed independently by two neuroradiologists experienced in neuro-oncologic MRI interpretation. In cases without visually increased perfusion, ROIs were placed within T2-FLAIR hyperintense tissue adjacent to the postoperative cavity. Maximum ASL signal intensity values measured from the lesion ROI were normalized separately to ROIs placed in the contralateral normal-appearing brain parenchyma and contralateral normal-appearing white matter at the corresponding symmetric anatomical location relative to the lesion, yielding relative signal intensity ratios (rSI^1^ and rSI^2^). Areas with increased ASL perfusion were considered suggestive of tumor recurrence. To improve reproducibility, care was taken to avoid obvious necrotic, cystic, hemorrhagic, or artifactual areas during ROI placement whenever possible.

DSC post-processing included identification of a local arterial input function and generation of perfusion maps. ROIs were placed in regions demonstrating the highest perfusion, avoiding necrotic or vascular structures. These measurements were also performed independently by the same two neuroradiologists. Cerebral blood volume (CBV) values measured from the lesion ROI were normalized separately to ROIs placed in the contralateral normal-appearing brain parenchyma and contralateral normal-appearing white matter at the corresponding symmetric anatomical location, yielding relative CBV ratios (rCBV^1^ and rCBV^2^).

Increased DSC perfusion was interpreted as indicative of tumor recurrence. Inter-reader reproducibility for continuous perfusion parameters was assessed statistically, and agreement for the final binary ASL- and DSC-based perfusion interpretations was also evaluated.

### 2.5. Reference Standard for Final Diagnosis

Histopathological confirmation was considered the reference standard when surgical resection or biopsy was performed within one month after the MRI examination. Histopathological verification was available for 10 of 63 MRI examinations, corresponding to 7 of 36 patients. However, performing surgery in follow-up patients is often not feasible. For examinations without histopathological verification, the final diagnosis was established based on clinical follow-up and serial MRI findings, reflecting routine postoperative neuro-oncologic practice in which repeat surgery or biopsy is not always feasible or clinically indicated. Lesions demonstrating progressive enlargement, increasing perfusion, or new enhancement on follow-up imaging on subsequent serial MRI examinations were classified as tumor recurrence, whereas lesions remaining stable or regressing over time without progressive perfusion changes were classified as treatment-related changes. Because radiation necrosis may occasionally show transient or variable enlargement mimicking tumor progression, final classification in non-histopathologically confirmed cases was not based on size increase on a single follow-up examination alone. Instead, serial imaging evolution, perfusion trends, enhancement pattern, and clinical follow-up were considered together. Lesions with transient enlargement followed by stabilization or regression without progressive perfusion increase were classified as treatment-related changes. In other words, final classification in non-histopathologically confirmed cases was based on the evolution of imaging findings over time rather than on a single follow-up examination. The minimum follow-up duration for examinations without histopathological confirmation was 3 months.

### 2.6. Exploratory Machine Learning Analysis

An exploratory machine learning (ML) analysis was conducted to further assess the diagnostic performance of perfusion MRI parameters in distinguishing tumor recurrence from treatment-related changes. Three predefined feature sets were evaluated separately: an ASL-only model (ASL_rSI1_mean, ASL_rSI2_mean), a DSC-only model (DSC_rCBV1_mean, DSC_rCBV2_mean), and a combined ASL–DSC model including all four variables. For each MRI examination, the input features were generated by averaging the corresponding measurements obtained by the two readers. Thus, the ML workflow consisted of feature generation, preprocessing, model training, grouped cross-validation, and performance evaluation.

A logistic regression classifier was used for all ML analyses. Logistic regression was selected as a relatively simple and interpretable classification algorithm appropriate for the limited number of predefined perfusion features used in this study. Feature standardization was performed using StandardScaler within a pipeline, ensuring that scaling was learned only from the training data within each fold and then applied to the corresponding test fold, thereby minimizing data leakage. To account for class imbalance, the logistic regression model was implemented with class_weight = “balanced”. In the combined ASL–DSC model, equal weights were not manually assigned to the four input variables. Instead, all four standardized perfusion features were entered simultaneously into the logistic regression model, and their coefficients were learned from the training data within each cross-validation fold. Thus, feature weighting was determined by the model during training rather than predefined by the investigators.

Model performance was evaluated using five-fold patient-level grouped cross-validation. Patient identifiers were used as grouping labels to ensure that multiple MRI examinations from the same patient were not split between the training and test sets within the same fold. This grouped approach was adopted to reduce within-patient dependency and provide a more realistic estimate of model generalizability in a longitudinal postoperative setting. Five-fold cross-validation was repeated multiple times, and performance metrics were reported as mean ± standard deviation. Evaluation metrics included Receiver Operating Characteristic—Area Under the Curve (ROC-AUC), precision–recall AUC (PR-AUC), sensitivity, and specificity. The evaluation metrics were defined as follows: accuracy = (TP + TN)/(TP + TN + FP + FN), sensitivity = TP/(TP + FN), and specificity = TN/(TN + FP), where TP, TN, FP, and FN denote true positives, true negatives, false positives, and false negatives, respectively. ROC-AUC was calculated as the area under the receiver operating characteristic curve, and PR-AUC as the area under the precision–recall curve.

To assess whether model performance could be attributed to chance, a permutation test was performed by randomly shuffling class labels 500 times while preserving the grouped cross-validation structure. The distribution of AUC values obtained from permuted labels was compared with the true-label model. This additional analysis was used to determine whether the observed classification performance was significantly greater than expected by chance.

### 2.7. Statistical Analysis

Continuous variables are presented as median (minimum–maximum) or mean ± standard deviation, as appropriate, and categorical variables as counts and percentages. The Shapiro–Wilk test was used to assess the normality of continuous variables. All statistical analyses, machine learning analyses, and visualizations were performed using Python (version 3.11.5), with the scipy, statsmodels, pandas, seaborn, matplotlib, and scikit-learn libraries.

Because the data did not follow a normal distribution, nonparametric tests were used. Differences between tumor recurrence and treatment-related changes were assessed using the Mann–Whitney U test for continuous variables. Agreement between perfusion-based imaging assessments and the final diagnosis was analyzed separately for qualitative and quantitative variables. For qualitative categorical assessments, including visual imaging interpretations, Cohen’s kappa was used to measure agreement with the reference standard diagnosis. Inter-reader agreement was also evaluated using Cohen’s kappa statistics. For continuous perfusion metrics, associations with the final diagnosis were evaluated through group comparisons and receiver operating characteristic (ROC)-based diagnostic performance analyses. Correlations between quantitative ASL and DSC perfusion parameters were analyzed using Spearman’s rank correlation coefficients.

Diagnostic performance metrics were calculated using standard definitions: accuracy as (TP + TN)/(TP + TN + FP + FN), sensitivity as TP/(TP + FN), specificity as TN/(TN + FP), positive predictive value as TP/(TP + FP), and negative predictive value as TN/(TN + FN), where TP, TN, FP, and FN denote true positive, true negative, false positive, and false negative results, respectively. ROC curve analysis was performed to assess diagnostic performance. Optimal cut-off values were determined using the Youden index. Sensitivity and specificity were calculated accordingly. A *p*-value < 0.05 was considered statistically significant.

## 3. Results

### 3.1. Patient Characteristics and Final Diagnosis

A total of 63 postoperative MRI examinations from patients with high-grade gliomas were included in the final analysis. The mean patient age was 51.3 ± 11.8 years, and 63.9% of the examinations were obtained from male patients. According to the final diagnosis based on longitudinal follow-up, 42 examinations (66.7%) were classified as treatment-related changes and 21 (33.3%) as tumor recurrence (Table 1).

### 3.2. Qualitative Assessment and Agreement with Reference Standard

**Figure 1:**
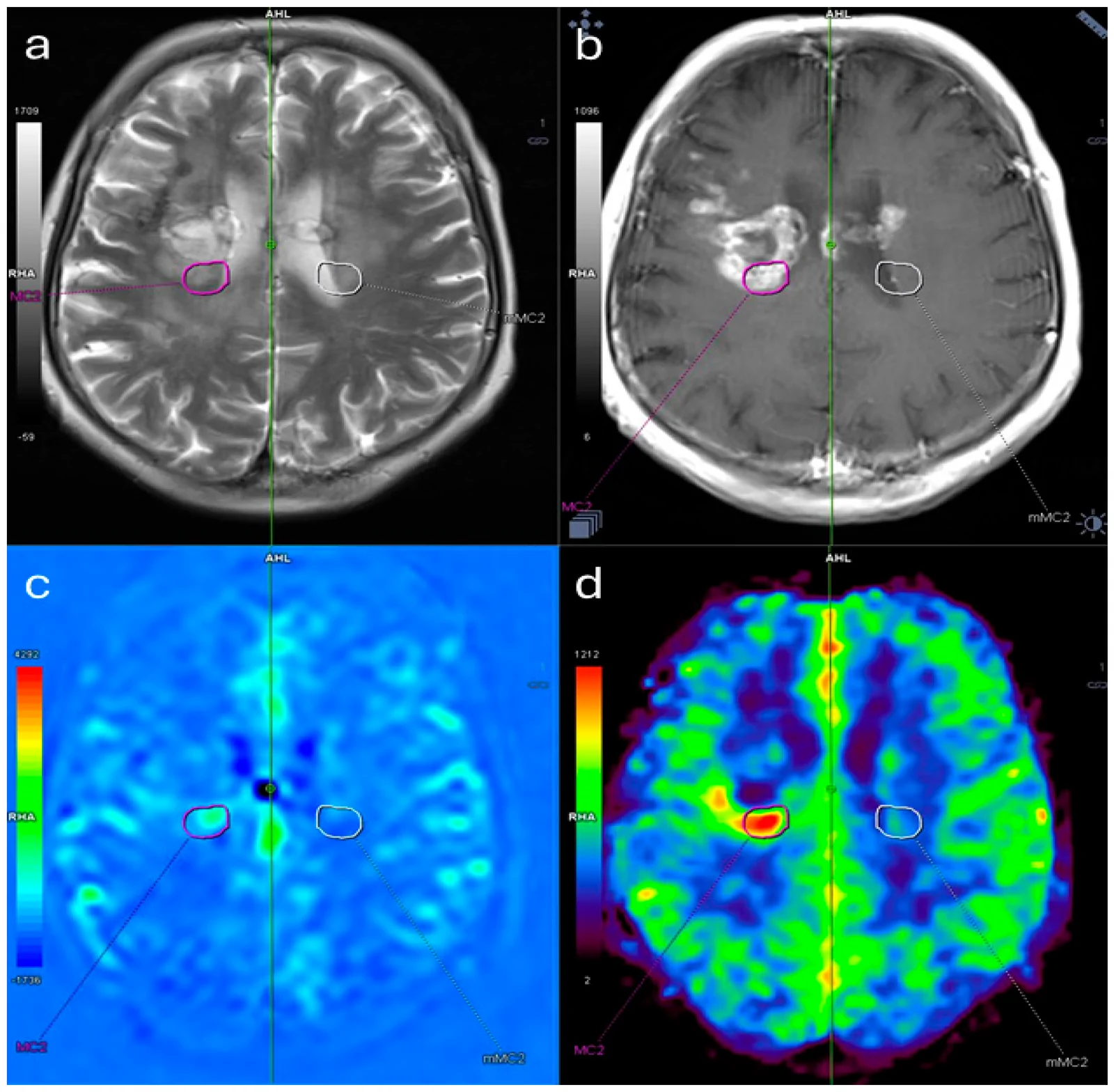
Tumor recurrence in a 64-year-old female patient with glioblastoma. Axial T2-weighted (**a**) and post-contrast T1-weighted (**b**) images show a lesion adjacent to the postoperative cavity. Arterial spin labeling (ASL) (**c**) and dynamic susceptibility contrast (DSC) (**d**) perfusion images demonstrate increased perfusion, consistent with tumor recurrence. Colored outlines indicate the lesion region of interest, and white outlines indicate the corresponding contralateral reference region used for comparison.

**Figure 2:**
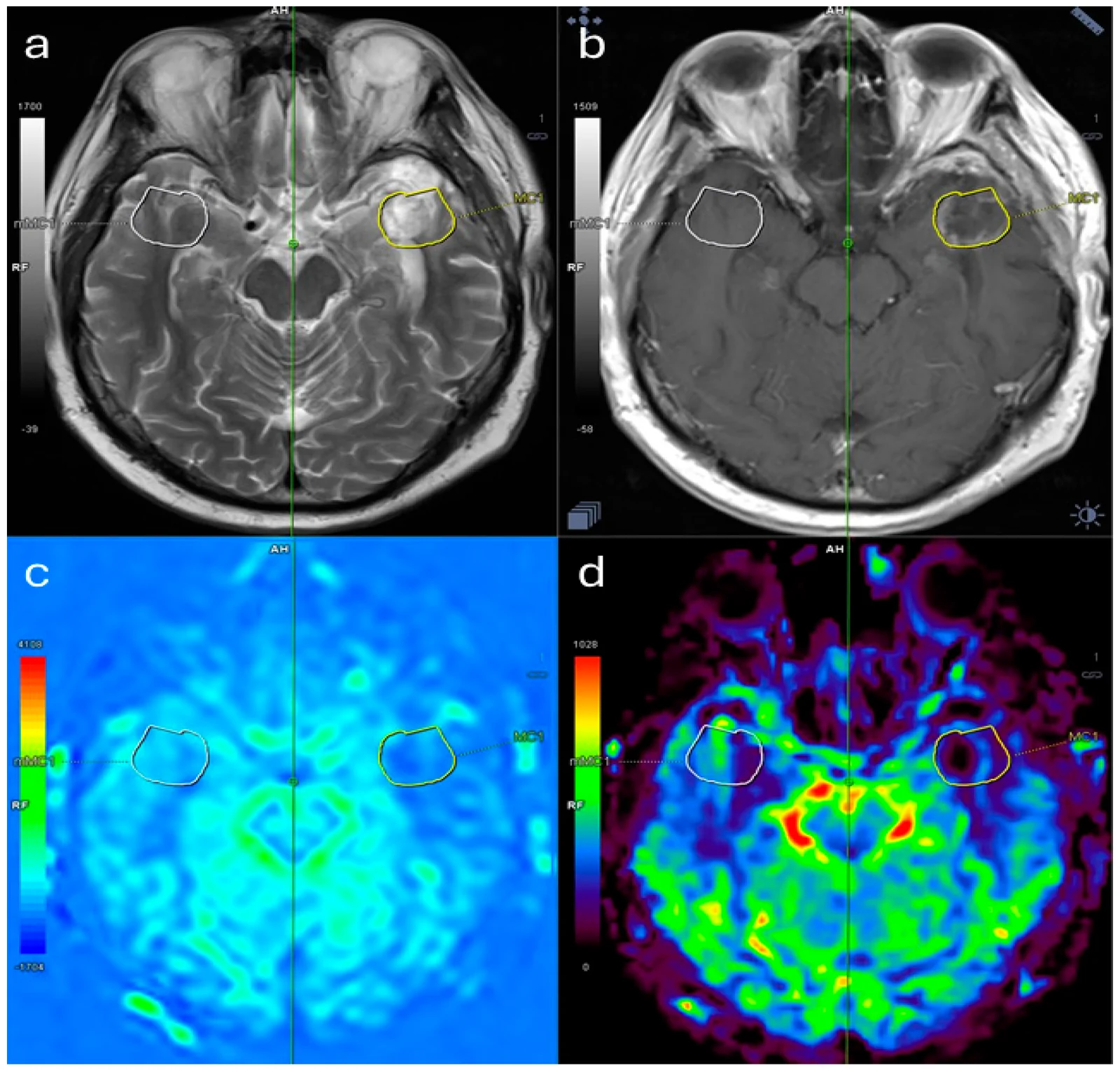
Treatment-related changes in a 58-year-old male patient with a history of glioblastoma. Axial T2-weighted (**a**) and post-contrast T1-weighted (**b**) images show postoperative changes without a mass-like lesion. Arterial spin labeling (ASL) (**c**) and dynamic susceptibility contrast (DSC) (**d**) perfusion images show no focal hyperperfusion. Colored outlines indicate the lesion region of interest, and white outlines indicate the corresponding contralateral reference region used for comparison.

Agreement between perfusion-based assessments and the final diagnosis is summarized in Table 2. For Reader 1, both ASL and DSC perfusion demonstrated excellent agreement with the final diagnosis (κ = 0.810 and κ = 0.850, respectively; *p* < 0.001 for both). Similarly, Reader 2 showed almost perfect agreement for both ASL and DSC perfusion (κ = 0.929 for both; *p* < 0.001), indicating high diagnostic consistency across readers. Representative examples of tumor recurrence and treatment-related changes based on ASL and DSC perfusion imaging are shown in Figure 1 and Figure 2.

### 3.3. Quantitative Perfusion Analysis

Quantitative comparisons of ASL-derived rSI and DSC-derived rCBV values between treatment-related changes and tumor recurrence are presented in Table 3. For both readers, all ASL rSI and DSC rCBV parameters were significantly higher in tumor recurrence compared with treatment-related changes (all *p* < 0.001). These findings were consistent across readers, confirming robust separation between the two diagnostic categories.

**Table 3:** Comparison of ASL-derived rSI values and DSC-derived rCBV values between treatment-related changes and tumor recurrence.

	Treatment-Related Changes	Tumor Recurrence	*p*-Value *
**Reader 1**			
ASL rSI ^1^	0.59 (0.14–1.38)	1.92 (0.39–3.71)	**<0.001**
ASL rSI ^2^	1.71 (0.40–4.04)	5.31 (0.64–22.22)	**<0.001**
DSC rCBV ^1^	0.81 (0.18–1.10)	1.73 (0.78–4.76)	**<0.001**
DSC rCBV ^2^	1.26 (0.51–1.76)	2.82 (1.35–9.85)	**<0.001**
**Reader 2**			
ASL rSI ^1^	0.64 (0.14–3.14)	2.86 (0.41–12.06)	**<0.001**
ASL rSI ^2^	1.11 (0.27–8.61)	5.68 (0.45–28.87)	**<0.001**
DSC rCBV ^1^	0.77 (0.29–2.01)	1.91 (1.05–3.89)	**<0.001**
DSC rCBV ^2^	1.35 (0.62–3.24)	3.00 (1.45–7.56)	**<0.001**

**Figure 3:**
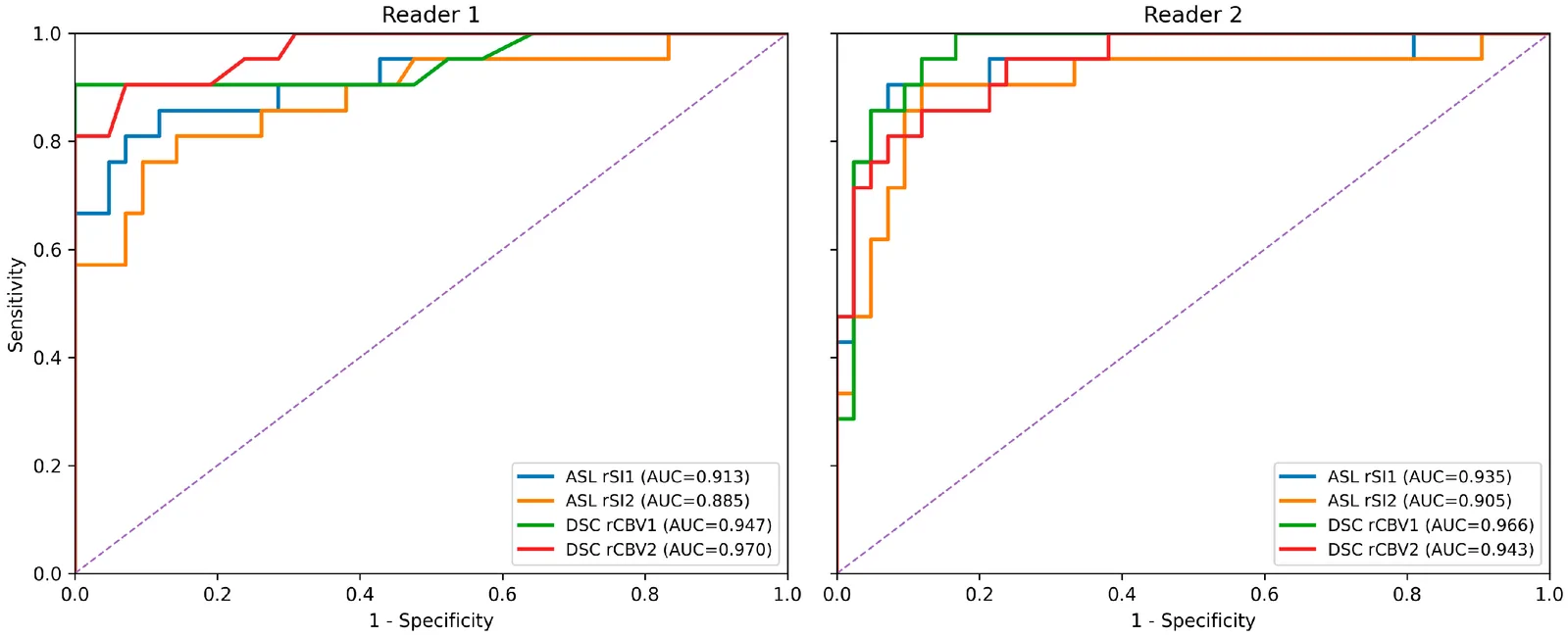
Receiver operating characteristic (ROC) curves showing the diagnostic performance of arterial spin labeling (ASL) and dynamic susceptibility contrast (DSC) perfusion parameters for differentiating tumor recurrence from treatment-related changes. Analyses are presented separately for Reader 1 (**left**) and Reader 2 (**right**).

* Mann–Whitney U test. Data are presented as median (min–max). Bold values indicate statistically significant differences. rSI ^1^ and rCBV ^1^ were normalized to contralateral normal-appearing brain parenchyma; rSI ^2^ and rCBV ^2^ were normalized to contralateral normal-appearing white matter. ROC analysis and diagnostic performance results are summarized in Table 4. For Reader 1, DSC-derived rCBV parameters achieved the highest AUC values, with a maximum AUC of 0.970 for rCBV2, while ASL rSI parameters also showed strong performance (AUC up to 0.913). For Reader 2, both ASL and DSC metrics yielded similarly high diagnostic accuracy, with AUC values reaching 0.966. Optimal cut-off values derived using the Youden index provided sensitivities ranging from 76.2% to 100.0% and specificities from 83.3% to 100.0%. Reader-based ROC analyses are presented in Figure 3.

**Table 4:** Diagnostic performance of ASL and DSC perfusion for tumor recurrence.

	AUC (95% CI)	Cut-Off	*p*-Value	Sensitivity	Specificity
**Reader 1**					
ASL perfusion	0.881 (0.780–0.982)		<0.001	76.2	100
ASL rSI ^1^	0.913 (0.825–1.000)	≥1.06	<0.001	81.0	92.9
ASL rSI ^2^	0.885 (0.785–0.984)	≥2.72	<0.001	81.0	85.7
DSC perfusion	0.905 (0.813–0.996)		<0.001	81.0	100
DSC rCBV ^1^	0.947 (0.878–1.000)	≥1.12	<0.001	90.5	100
DSC rCBV ^2^	0.970 (0.917–1.000)	≥1.56	<0.001	90.5	92.9
**Reader 2**					
ASL perfusion	0.964 (0.907–1.000)		<0.001	95.2	97.6
ASL rSI ^1^	0.935 (0.859–1.000)	≥1.25	<0.001	90.5	92.9
ASL rSI ^2^	0.905 (0.813–0.996)	≥2.32	<0.001	90.5	88.1
DSC perfusion	0.964 (0.907–1.000)		<0.001	95.2	97.6
DSC rCBV ^1^	0.966 (0.910–1.000)	≥1.05	<0.001	100.0	83.3
DSC rCBV ^2^	0.943 (0.872–1.000)	≥1.99	<0.001	81.0	92.9

Cut-off values were determined using the Youden index. AUC: area under the ROC curve; CI: confidence interval. rSI ^1^ and rCBV ^1^ were normalized to contralateral normal-appearing brain parenchyma; rSI ^2^ and rCBV ^2^ were normalized to contralateral normal-appearing white matter.

### 3.4. Inter-Reader Reproducibility and Agreement

Inter-reader correlation analysis revealed moderate to strong correlations between Reader 1 and Reader 2 for all continuous perfusion parameters (Table 5). Spearman’s rho values ranged from 0.583 to 0.704 (all *p* < 0.001), indicating good reproducibility of quantitative ASL and DSC measurements between readers.

Finally, inter-reader agreement for binary ASL and DSC perfusion-based assessments is shown in Table 6. Agreement between readers was excellent for both ASL (κ = 0.810, *p* < 0.001) and DSC perfusion (κ = 0.850, *p* < 0.001), demonstrating substantial to almost perfect inter-reader reliability.

### 3.5. Machine Learning-Based Classification Analysis

**Figure 4:**
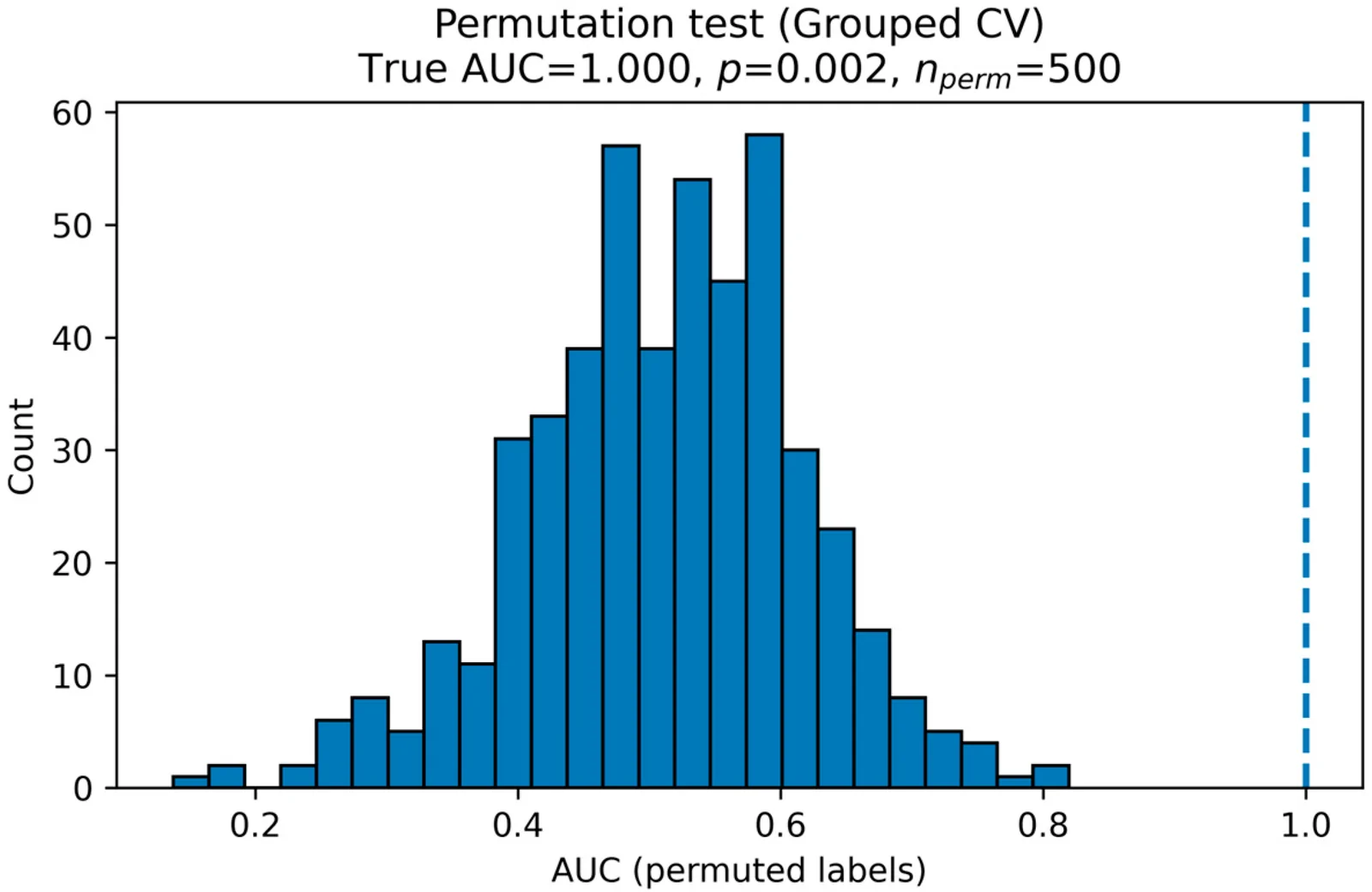
Permutation test for the combined arterial spin labeling–dynamic susceptibility contrast machine learning model using patient-level grouped cross-validation. The histogram shows the distribution of area under the curve values obtained from permuted class labels (*n* = 500). The dashed vertical line indicates the true model performance.

In a patient-level grouped cross-validation analysis, logistic regression models based on ASL-only, DSC-only, and combined perfusion parameters demonstrated good to excellent diagnostic performance (Table 7). The combined model achieved the highest mean AUC. Permutation testing using patient-level grouped cross-validation demonstrated that the observed performance of the combined model was unlikely to be due to chance, with the true AUC lying well outside the null distribution obtained from permuted labels (empirical *p* = 0.002). Permutation testing confirmed that the performance of the combined ASL–DSC machine learning model was significantly higher than chance (Figure 4).

## 4. Discussion

In this study, we investigated the diagnostic performance of a non-contrast perfusion approach in patients with surgically treated high-grade gliomas. Our findings demonstrate that when non-contrast conventional MRI sequences are evaluated in combination with ASL perfusion imaging, tumor recurrence can be detected with diagnostic performance comparable to that of contrast-enhanced MRI and DSC perfusion imaging. In our cohort, both qualitative and quantitative analyses demonstrated that ASL perfusion achieved high diagnostic accuracy in differentiating tumor recurrence from treatment-related changes. ROC analysis revealed that ASL-derived parameters yielded high AUC values for both readers, approaching those obtained with DSC perfusion. Notably, ASL perfusion showed high specificity across readers, supporting its potential role as a reliable non-contrast alternative in postoperative surveillance.

Radiological findings in patients with brain tumors vary substantially depending on the timing of imaging and the type of treatment administered. Surgical resection represents the primary therapeutic approach following the initial diagnosis of glioma and is also frequently performed in cases of tumor recurrence. Surgical strategies range from minimally invasive biopsy to extensive craniotomy, with the goal of achieving maximal safe resection while preserving neurological function. Despite advances in surgical techniques, conventional MRI sequences alone remain insufficient for reliably distinguishing treatment-related changes—such as radiation necrosis, pseudoprogression, and pseudoregression—from true tumor recurrence. Consequently, advanced imaging modalities, including perfusion MRI, diffusion tensor imaging, and MR spectroscopy, have become integral components of post-treatment imaging assessment [1,3,11].

Perfusion MRI enables the evaluation of tissue vascularity and hemodynamics and can be performed using three main techniques: DSC and DCE perfusion MRI, which require exogenous contrast agents, and ASL, which relies on an endogenous contrast mechanism. DSC perfusion is based on T2*-weighted signal loss caused by gadolinium-induced susceptibility effects, whereas ASL uses magnetically labeled arterial blood water as a freely diffusible tracer. Although DSC perfusion remains the most widely used technique in clinical practice, ASL has gained increasing attention due to its non-contrast nature. ASL perfusion generates a flow-labeled image and a control image in which static tissue signals are identical, allowing cerebral blood flow to be quantified from their subtraction [5]. Inter-reader agreement analysis demonstrated substantial to almost perfect agreement for both ASL and DSC perfusion assessments. The high concordance between readers suggests that ASL perfusion measurements are reproducible and interpretable in routine clinical practice, despite previously reported concerns regarding variability and signal-to-noise limitations.

Despite its advantages, ASL is associated with several technical limitations, most notably its relatively low signal-to-noise ratio and longer acquisition times compared with DSC perfusion. Typical ASL acquisitions last between 5 and 10 min; in our study, the acquisition time was approximately 4 min and 59 s. Longer acquisition times may compromise image quality due to motion and physiological variability. Additionally, many ASL implementations rely on echo-planar imaging, which is susceptible to geometric distortion in regions with high magnetic field inhomogeneity, particularly at the skull base. ASL and DSC perfusion MRI are not equally susceptible to location-related artifacts, because their artifact profiles differ according to acquisition principles. DSC perfusion, which relies on T2*-weighted dynamic signal changes after gadolinium administration, may be particularly vulnerable to susceptibility artifacts near bone–air interfaces, postoperative blood products, calcifications, hemorrhage, and surgical materials. In addition, contrast leakage may affect DSC-derived CBV measurements in enhancing tumors. ASL, although not affected by gadolinium leakage and not requiring contrast injection, may still be influenced by low signal-to-noise ratio, patient motion, arterial transit delay effects, and geometric distortion related to EPI- or GRASE-based readouts, especially in lesions close to the skull base. Therefore, in clinical interpretation, lesion location and local postoperative changes should be considered when comparing ASL and DSC perfusion findings. Recent developments—including the use of rapid 3D acquisition techniques and high-field-strength MRI—have substantially improved ASL image quality by increasing signal-to-noise ratio and prolonging T1 relaxation times [12,13,14,15,16]. In this context, the use of a 3 Tesla scanner and a 3D pulsed ASL technique represents a significant technical strength of the present study.

ASL has been successfully applied in a wide range of clinical scenarios, including ischemic stroke, steno-occlusive disease, neurodegenerative disorders, epilepsy, and brain tumors [17]. In neuro-oncology, ASL has demonstrated value not only in preoperative tumor grading but also in postoperative follow-up, particularly for distinguishing residual or recurrent tumor from treatment-related changes such as radiation necrosis and pseudoprogression [18,19,20,21,22,23]. Razek et al. reported significant differences in cerebral blood flow values between residual or recurrent glioblastoma and post-treatment changes using ASL, highlighting its potential clinical utility [22]. Similarly, Ye et al. demonstrated a strong linear correlation between ASL and DSC perfusion parameters in differentiating tumor recurrence from radiation injury [18]. Xu et al. compared pseudo-continuous ASL with DSC perfusion and reported higher sensitivity but lower specificity for ASL-derived perfusion metrics [24]. Bayraktar et al. found that while both ASL and DSC perfusion were diagnostically useful, the highest accuracy was achieved using DSC-derived rCBV values [25].

In contrast to previous studies that primarily focused on direct comparisons between ASL and DSC perfusion metrics, the present study aimed to enhance the diagnostic effectiveness of ASL by integrating its findings with non-contrast conventional MRI sequences during image interpretation. Notably, our results demonstrated that ASL perfusion achieved higher specificity than DSC perfusion for both readers, suggesting that a carefully interpreted non-contrast imaging protocol may provide robust diagnostic confidence in selected patient populations.

Several ASL strategies have been described, including continuous ASL, pulsed ASL, pseudo-continuous ASL, and velocity-selective ASL, each with distinct technical advantages and limitations. Pulsed ASL, as used in this study, employs short radiofrequency pulses for labeling and offers ease of implementation with reduced technical complexity compared with continuous ASL [26,27]. Pseudo-continuous ASL combines favorable features of both continuous and pulsed techniques, while velocity-selective ASL eliminates transit delay effects by labeling spins based on flow velocity [16]. The choice of pulsed ASL in our protocol reflects a balance between technical feasibility and clinical applicability.

In an exploratory ML analysis using patient-level grouped cross-validation, models based on ASL-only, DSC-only, and combined perfusion parameters demonstrated consistent diagnostic performance. Notably, ASL-based models achieved robust classification accuracy, supporting the ability of non-contrast perfusion metrics to capture clinically meaningful hemodynamic differences. While the combined ASL–DSC model yielded the highest overall performance, the strong results obtained with ASL alone further emphasize its potential utility in reducing reliance on contrast-enhanced imaging during follow-up. While the DSC-only and combined models yielded near-perfect AUC values, these findings should be interpreted cautiously. Although such high performance may raise concern for overfitting, several design features reduce this risk, including the use of patient-level grouped cross-validation, a simple regularized logistic regression model, and a limited number of predefined input features. In addition, permutation testing preserving the grouped structure demonstrated that the observed performance was significantly higher than expected by chance (empirical *p* = 0.002). Nevertheless, because this was a single-center study with a relatively limited sample size, the ML findings should be considered exploratory and require validation in larger independent cohorts.

This study has several limitations. First, its single-center design and relatively small sample size may limit generalizability. Second, histopathological confirmation was not available for all cases, particularly among patients classified as having treatment-related changes. In addition, even histopathological verification may be affected by sampling error in heterogeneous post-treatment lesions, because small foci of viable tumors may coexist with radiation necrosis or other treatment-related changes and may be missed by biopsy. This limitation further supports the use of longitudinal clinical and imaging follow-up as part of the reference standard. Third, complete and systematically verified overall survival data were not available for all patients; therefore, we could not evaluate the relationship between ASL- or DSC-derived perfusion intensity and survival outcomes. Future studies with larger cohorts and longitudinal survival data are warranted to determine whether ASL or DSC perfusion parameters provide stronger prognostic information in patients with high-grade glioma. The relatively small cohort and limited recruitment period also represent important limitations; larger multicenter studies with longer recruitment periods are needed to validate these findings. In current clinical practice, ASL should not be interpreted as a universal replacement for standard post-contrast MRI or DSC perfusion. Rather, ASL may serve as a complementary non-contrast perfusion technique in routine follow-up and may be particularly useful when gadolinium administration is contraindicated or undesirable. Nevertheless, all imaging examinations were acquired using a standardized protocol on the same 3 Tesla MRI system, and image interpretation was performed independently by two experienced neuroradiologists, which strengthens the internal consistency of the findings.

## 5. Conclusions

This study demonstrates, through a machine learning-based approach, that non-contrast ASL perfusion achieves diagnostic performance comparable to the clinical standard of DSC-MRI in distinguishing high-grade glioma recurrence from treatment effects. While our findings indicate that a combined ASL-DSC model yields the highest diagnostic accuracy, ASL-derived metrics independently provide robust classification with high specificity. Consequently, ASL represents not merely a substitute, but a reliable and effective non-invasive alternative for longitudinal surveillance, particularly for patients in whom gadolinium-based contrast is contraindicated or where cumulative contrast retention is a concern.

## Figures and Tables

**Table 1:** Patient characteristics, reader-based qualitative perfusion assessments, and final diagnosis.

Age (years) *	51.25 ± 11.78
**Gender**	
Female	13 (36.1%)
Male	23 (63.9%)
**Reader 1**	
**ASL perfusion**	
Treatment-related changes	47 (74.6%)
Tumor recurrence	16 (25.4%)
**DSC perfusion**	
Treatment-related changes	46 (73.0%)
Tumor recurrence	17 (27.0%)
**Reader 2**	
**ASL perfusion**	
Treatment-related changes	42 (66.7%)
Tumor recurrence	21 (33.3%)
**DSC perfusion**	
Treatment-related changes	42 (66.7%)
Tumor recurrence	21 (33.3%)
**Final diagnosis**	
Treatment-related changes	42 (66.7%)
Tumor recurrence	21 (33.3%)

* Data are presented as mean ± standard deviation.

**Table 2:** Agreement of ASL and DSC perfusion findings with final diagnosis in differentiating tumor recurrence from treatment-related changes.

	Treatment-Related Changes	Tumor Recurrence	Kappa	*p*-Value *
**Reader 1**				
**ASL perfusion**				
Treatment-related changes	42 (100.0)	0 (0.0)	0.810	<0.001
Tumor recurrence	5 (23.8)	16 (76.2)		
**DSC perfusion**				
Treatment-related changes	42 (100.0)	0 (0.0)	0.85	<0.001
Tumor recurrence	4 (19.0)	17 (81.0)		
**Reader 2**				
**ASL perfusion**				
Treatment-related changes	41 (97.6)	1 (2.4)	0.929	<0.001
Tumor recurrence	1 (4.8)	20 (95.2)		
**DSC perfusion**				
Treatment-related changes	41 (97.6)	1 (2.4)	0.929	<0.001
Tumor recurrence	1 (4.8)	20 (95.2)		

* *p*-values were calculated using Cohen’s kappa test. Data are presented as counts with row percentages in brackets.

**Table 5:** Inter-reader correlation of ASL- and DSC-derived perfusion parameters.

Reader 1–Reader 2	rs	*p*-Value
ASL rSI ^1^	0.704	**<0.001**
ASL rSI ^2^	0.619	**<0.001**
DSC rCBV ^1^	0.611	**<0.001**
DSC rCBV ^2^	0.583	**<0.001**

Bold values indicate statistically significant differences. rs: Spearman’s rho correlation coefficient. rSI ^1^ and rCBV ^1^ were normalized to contralateral normal-appearing brain parenchyma; rSI ^2^ and rCBV ^2^ were normalized to contralateral normal-appearing white matter.

**Table 6:** Inter-reader agreement of ASL and DSC perfusion-based assessments.

	ASL Perfusion (Reader 2)		Kappa	*p*-Value *
	Treatment-Related Changes	Tumor Recurrence		
**ASL perfusion (Reader 1)**				
Treatment-related changes	42 (89.4)	5 (10.6)	0.810	<0.001
Tumor recurrence	0 (0.0)	16 (100.0)		
	**DSC perfusion (Reader 2)**			
	Treatment-related changes	Tumor recurrence		
**DSC perfusion (Reader 1)**				
Treatment-related changes	42 (91.3)	4 (8.7)	0.850	<0.001
Tumor recurrence	0 (0.0)	17 (100.0)		

* *p*-values were calculated using Cohen’s kappa test was used to assess inter-reader agreement. Data are presented as counts, with row percentages in parentheses.

**Table 7:** Performance of patient-level logistic regression models in grouped cross-validation.

Model	ROC-AUC (Mean ± SD)	PR-AUC (Mean ± SD)	Accuracy (Mean ± SD)	Sensitivity (Mean ± SD)	Specificity (Mean ± SD)
ASL-only	0.946 ± 0.089	0.953 ± 0.075	0.943 ± 0.080	0.913 ± 0.154	0.979 ± 0.059
DSC-only	0.997 ± 0.010	0.995 ± 0.017	0.992 ± 0.023	0.993 ± 0.034	0.994 ± 0.022
Combined	0.997 ± 0.008	0.995 ± 0.017	0.993 ± 0.023	0.997 ± 0.023	0.991 ± 0.031

## Data Availability

The data presented in this study are available on request from the corresponding author. The data are not publicly available due to privacy and ethical restrictions.

## Abbreviations

The following abbreviations are used in this manuscript:

AUC	Area under the curve
ASL	Arterial spin labeling
CBV	Cerebral blood volume
DSC	Dynamic susceptibility contrast
ML	Machine learning
PR-AUC	Precision–recall area under the curve
rCBV	Relative cerebral blood volume
rSI	Relative signal intensity
ROC	Receiver operating characteristic
ROI	Region of interest

## References

[1] Martucci M., Russo R., Giordano C., Schiarelli C., D’Apolito G., Tuzza L., Lisi F., Ferrara G., Schimperna F., Vassalli S. (2023). Advanced Magnetic Resonance Imaging in the Evaluation of Treated Glioblastoma: A Pictorial Essay. Cancers.

[2] Momeni F., Abedi-Firouzjah R., Farshidfar Z., Taleinezhad N., Ansari L., Razmkon A., Banaei A., Mehdizadeh A. (2021). Differentiating Between Low- and High-grade Glioma Tumors Measuring Apparent Diffusion Coefficient Values in Various Regions of the Brain. Oman Med. J..

[3] Horbinski C., Nabors L.B., Portnow J., Baehring J., Bhatia A., Bloch O., Brem S., Butowski N., Cannon D.M., Chao S. (2023). NCCN guidelines® insights: Central nervous system cancers, version 2.2022: Featured updates to the NCCN guidelines. J. Natl. Compr. Cancer Netw..

[4] Provenzale J.M., Mukundan S., Barboriak D.P. (2006). Diffusion-weighted and perfusion MR imaging for brain tumor characterization and assessment of treatment response. Radiology.

[5] Williams D.S., Detre J.A., Leigh J.S., Koretsky A.P. (1992). Magnetic resonance imaging of perfusion using spin inversion of arterial water. Proc. Natl. Acad. Sci. USA.

[6] Kuo P.H., Kanal E., Abu-Alfa A.K., Cowper S.E. (2007). Gadolinium-based MR contrast agents and nephrogenic systemic fibrosis. Radiology.

[7] Hirai T., Kitajima M., Nakamura H., Okuda T., Sasao A., Shigematsu Y., Utsunomiya D., Oda S., Uetani H., Morioka M. (2011). Quantitative blood flow measurements in gliomas using arterial spin-labeling at 3T: Intermodality agreement and inter-and intraobserver reproducibility study. Am. J. Neuroradiol..

[8] Nael K., Meshksar A., Liebeskind D.S., Coull B.M., Krupinski E.A., Villablanca J.P. (2013). Quantitative analysis of hypoperfusion in acute stroke: Arterial spin labeling versus dynamic susceptibility contrast. Stroke.

[9] Chen J., Zhao B., Bu C., Xie G. (2014). Relationship between the hemodynamic changes on multi-Td pulsed arterial spin labeling images and the degrees of cerebral artery stenosis. Magn. Reson. Imaging.

[10] Mak H.K., Qian W., Ng K.S., Chan Q., Song Y.Q., Chu L.W., Yau K.K. (2014). Combination of MRI hippocampal volumetry and arterial spin labeling MR perfusion at 3-Tesla improves the efficacy in discriminating Alzheimer’s disease from cognitively normal elderly adults. J. Alzheimer’s Dis. JAD.

[11] Weller M., van den Bent M., Preusser M., Le Rhun E., Tonn J.C., Minniti G., Bendszus M., Balana C., Chinot O., Dirven L. (2021). EANO guidelines on the diagnosis and treatment of diffuse gliomas of adulthood. Nat. Rev. Clin. Oncol..

[12] Razek A., El-Serougy L., Abdelsalam M., Gaballa G., Talaat M. (2018). Differentiation of residual/recurrent gliomas from postradiation necrosis with arterial spin labeling and diffusion tensor magnetic resonance imaging-derived metrics. Neuroradiology.

[13] Griffith B., Jain R. (2015). Perfusion imaging in neuro-oncology: Basic techniques and clinical applications. Radiol. Clin..

[14] Haller S., Zaharchuk G., Thomas D.L., Lovblad K.-O., Barkhof F., Golay X. (2016). Arterial spin labeling perfusion of the brain: Emerging clinical applications. Radiology.

[15] Havsteen I., Damm Nybing J., Christensen H., Christensen A.F. (2018). Arterial spin labeling: A technical overview. Acta Radiol..

[16] Petcharunpaisan S., Ramalho J., Castillo M. (2010). Arterial spin labeling in neuroimaging. World J. Radiol..

[17] Jaafar N., Alsop D.C. (2024). Arterial Spin Labeling: Key Concepts and Progress Towards Use as a Clinical Tool. Magn. Reson. Med. Sci. MRMS Off. J. Jpn. Soc. Magn. Reson. Med..

[18] Ye J., Bhagat S.K., Li H., Luo X., Wang B., Liu L., Yang G. (2016). Differentiation between recurrent gliomas and radiation necrosis using arterial spin labeling perfusion imaging. Exp. Ther. Med..

[19] Nyberg E., Honce J., Kleinschmidt-DeMasters B.K., Shukri B., Kreidler S., Nagae L. (2016). Arterial spin labeling: Pathologically proven superiority over conventional MRI for detection of high-grade glioma progression after treatment. Neuroradiol. J..

[20] Fu J., Li L., Wang X., Zhang M., Zhang Y., Li Z. (2018). Clinical utility of arterial spin labeling for preoperative grading of glioma. Biosci. Rep..

[21] Ningning D., Haopeng P., Xuefei D., Wenna C., Yan R., Jingsong W., Chengjun Y., Zhenwei Y., Xiaoyuan F. (2017). Perfusion imaging of brain gliomas using arterial spin labeling: Correlation with histopathological vascular density in MRI-guided biopsies. Neuroradiology.

[22] Razek A.A.K.A., Talaat M., El-Serougy L., Gaballa G., Abdelsalam M. (2019). Clinical applications of arterial spin labeling in brain tumors. J. Comput. Assist. Tomogr..

[23] Cebeci H., Aydin O., Ozturk-Isik E., Gumus C., Inecikli F., Bekar A., Kocaeli H., Hakyemez B. (2014). Assesment of perfusion in glial tumors with arterial spin labeling; comparison with dynamic susceptibility contrast method. Eur. J. Radiol..

[24] Xu Q., Liu Q., Ge H., Ge X., Wu J., Qu J., Xu K. (2017). Tumor recurrence versus treatment effects in glioma: A comparative study of three dimensional pseudo-continuous arterial spin labeling and dynamic susceptibility contrast imaging. Medicine.

[25] Bayraktar E.S., Duygulu G., Çetinoğlu Y.K., Gelal M.F., Apaydın M., Ellidokuz H. (2024). Comparison of ASL and DSC perfusion methods in the evaluation of response to treatment in patients with a history of treatment for malignant brain tumor. BMC Med. Imaging.

[26] Wu W.C., Fernández-Seara M., Detre J.A., Wehrli F.W., Wang J. (2007). A theoretical and experimental investigation of the tagging efficiency of pseudocontinuous arterial spin labeling. Magn. Reson. Med..

[27] Petersen E.T., Lim T., Golay X. (2006). Model-free arterial spin labeling quantification approach for perfusion MRI. Magn. Reson. Med..

